# Extraction and characterization of a novel glycosylated naphthazarin pigment from mangrove *Aspergillus unguis* AUMC15225

**DOI:** 10.1038/s41598-026-43500-0

**Published:** 2026-04-01

**Authors:** Basma M. Alkersh, Hanan A. Ghozlan, Soraya A. Sabry, Sahar W. M. Hassan, Amany El-Sikaily

**Affiliations:** 1 National Institute of Oceanography and Fisheries (NIOF), Cairo, Egypt; 2 Faculty of Science, Alexandria University, Alexandria, Egypt

**Keywords:** *Aspergillus*, Mangrove, Epiphytic, Natural pigments, Secondary metabolites, Naphthazarin, Biochemistry, Biotechnology, Chemistry, Microbiology, Plant sciences

## Abstract

Natural naphthoquinone (NQ) dyes are gaining attention as safer and more sustainable alternative to synthetic colorants, and their glycosylation can enhance solubility and stability. In the present study, a novel, extracellular, highly stable, glycosylated derivative of NQ pigments (2-O-β-L-arabinofuranosyl-5,8-dihydroxy-1,4-naphthoquinone, C₁₅H₁₆O₈) was produced by the mangrove epiphytic fungus, *Aspergillus unguis*. Pigment production kinetics and solubility testing indicated the secondary metabolic behavior and high polarity of the pigment. HPLC separated the pigment into two isomeric fractions with retention times of 4.840 and 6.020 min. Structural characterization was supported by several analyses, with a λ_max_ at 300 nm and R_f_ values of 0.76–0.8. Furthermore, FTIR spectroscopy has revealed the key peaks of naphthazarin core at 1642, 1255–1262, 3331–3335, 1650, 918, 700, and 553 cm⁻1. In addition, glycosylation characteristic peaks were found at 3396, 1200–1260, 1144, 1101, and 1028 cm⁻1. Quinone carbonyls and α hydroxyls’ hydrogen bonding in the naphthazarin core was indicated by the peaks at 2920–2933 cm^-1^. The molecular weight of the neutral compound was found to be 324 Da via LCMS. NMR spectroscopy has displayed characteristic signals confirming the proposed structure. Proton NMR showed signals at δ 6.586 and 6.214 ppm (aromatic protons: H-6 and H-3 of the naphthazarin core); 4.892 ppm (anomeric proton H-1΄ of the sugar moiety); 4.85–4.22 ppm (anomeric protons of the pentose sugar). Moreover, ^13^C NMR displayed signals at δ 97.38 ppm (anomeric carbon C-1΄), 77–70 ppm (oxygenated sugar carbons of pentose), 61.61 ppm (hydroxymethyl carbon C-5΄ that confirms the furanose structure).

**Supplementary Information:**

The online version contains supplementary material available at 10.1038/s41598-026-43500-0.

## Introduction

Mangrove ecosystems are important reservoirs of unique microbial communities that produce diverse bioactive metabolites, because of their adaptation to extreme environmental conditions. Among these microorganisms, mangrove-associated fungi exhibit distinctive metabolic capabilities linked to survival in these habitats^1^. They are considered an underexplored reservoir of natural products with diverse biological activities and novel chemical structures^1,2^. These metabolites have potential applications in medical, biotechnological, agricultural, and industrial fields^3^.

One class of compounds that has recently attracted considerable attention is natural pigments. This interest originates from their role as sustainable alternatives to synthetic dyes^4^, which are often toxic, environmentally hazardous, and restricted in several regions, including the EU, India, and the United States. Within natural pigments, quinones—particularly anthraquinones, naphthoquinones (NQs), and benzoquinones—are important because of their structural similarity to many synthetic dyes. NQs are aromatic compounds consisting of a fused benzene and quinone ring system derived from naphthalene and two carbonyl groups on one ring, enabling extensive electron delocalization that causes their characteristic coloration. These compounds typically occur as glycosylated derivatives in nature^5^, predominantly in plants and, to a lesser extent, in fungi, bacteria, and animals^6^.

NQs’ glycosylation is reported to enhance their water solubility, which improves bioavailability, selectivity, stability, and overall biological activity^7^. Despite growing interest in natural pigments as sustainable and safer alternatives to synthetic dyes, glycosylated naphthoquinones remain underexplored, particularly those derived from mangrove-associated fungi. Shen, et al.^5^ have reported that glycosylation of NQs, such as lapachol and lawson, can augment immunomodulatory, anticancer, antibacterial activities by modulating molecular interactions and redox behavior, reactive oxygen species (ROS)-mediated signaling, and reducing nonspecific cytotoxicity. These derivatives can be synthesized intracellularly via glycosyltransferase enzymes, such as rhamnopyranoside-plumbagin from *Plumbago indica*^8^. Although research on glycosylated NQs remains limited, their structural diversity and stereochemical features suggest considerable potential for drug discovery^5^. To the best of our knowledge, no biological or synthetic production method has yet been reported for an arabinofuranose-conjugated NQ, a structure that also remains undocumented in the literature. Therefore, green production and characterization of such a novel metabolite is necessary for unlocking its application potential.

To the best of our knowledge, this study reports the first production, and structural characterization of an arabinofuranose-conjugated naphthoquinone pigment. It’s biosynthesized by *Aspergillus unguis* AUMC15225 which is an epiphytic fungus isolated from mangrove aerial roots. In addition to stability assessment and physicochemical characterization, structural elucidation was performed using UV–visible spectroscopy (UV–vis), high-performance liquid chromatography (HPLC), thin-layer chromatography (TLC), Fourier-transform infrared spectroscopy (FTIR), liquid chromatography–mass spectrometry (LC–MS), and nuclear magnetic resonance (NMR) spectroscopy. Overall, this work highlights mangrove-associated fungi as a promising source of novel natural pigments with unique structural features and potential biotechnological relevance.

## Materials and methods

### Strains, media and culture conditions

*Aspergillus unguis* AUMC15225 is an epiphytic fungal strain isolated from Hidden Bay, mangrove aerial roots, Ras Mohammad Natural Reserve, Red Sea (27.7383° N, 34.2427° E). Sampling was conducted in November 2019, during normal tidal conditions in the intertidal mangrove zone, with ambient temperatures of approximately 29 °C. The fungus was routinely sub-cultured on Sabouraud Dextrose Agar (SDA; Difco®) and preserved at the Assiut University Mycological Center (AUMC), Egypt. SDA was prepared according to the manufacturer’s instructions, and the cultures were incubated at 25 °C for 15 days.

For pigment production in both shaken and static incubation, Sabouraud Dextrose Broth (SDB; Difco®) was prepared using 50% seawater (salinity = 39 ppt). A 100 mL SDB flask was inoculated with 1 mL of spore suspension containing 10⁶ spores/mL of *A. unguis* AUMC15225 and incubated at 25 °C for 18 days.

### Solvents and chemicals

All solvents used for pigment extraction, purification, solubility testing, chromatographic separation, and spectroscopic analyses were of analytical or HPLC grade. n-Hexane (≥ 98%), ethyl acetate (≥ 99.8%), dichloromethane (≥ 99.9%), n-propanol (≥ 98%), methanol (≥ 99.85%), ethanol (99.9%), petroleum ether (60–90 °C), chloroform (≥ 99.8%), acetone (≥ 99.8%), n-butanol (≥ 99.8%), acetonitrile (HPLC grade, ≥ 99.9%), and formic acid (≥ 98%) were purchased from Merck® (Darmstadt, Germany). Dimethyl sulfoxide-d₆ (DMSO-d₆, 99.95 atom % D) used for NMR analysis was from Merck®.

All chemicals and reagents used for media preparation, phytochemical screening, and analytical procedures, including sodium hydroxide (NaOH), hydrochloric acid (HCl), potassium bromide (KBr), and p-anisaldehyde, were of analytical grade and supplied by Sigma-Aldrich® (St. Louis, MO, USA). Deionized water was used throughout the study.

### Culture fermentation for pigment production

For pigment production, 1 mL of a freshly prepared fungal spores’ suspension (10^6^ spores/mL) was used to inoculate a 100 mL SDB (50% seawater) flask and incubated at 25 °C till maximum growth and maximum pigment production. A sterile SDB flask was incubated under the same conditions and used as a negative control. At the end of incubation, filtration using Whatman Grade 1 qualitative filter paper (110 mm diameter) was performed to separate the mycelial mat from the filtrate under aseptic conditions. Maximum absorbance (λ_max_) of diluted filtrate (1:10) was measured at 200–700 nm UV–vis by spectrophotometer (Helios Alpha, 9423 UVA 1002E spectrophotometer)^9^.

### Pigment production kinetics

One milliliter of a fungal spore suspension (10⁶ spores/mL) was inoculated into 100 mL of SDB medium prepared with 50% seawater, and the cultures were incubated at 25 °C for 21 days. A sterile, uninoculated SDB flask maintained under the same conditions served as the negative control. All tests were performed in triplicate, and results were expressed as mean ± Standard deviation (SD). Percentage error bars were used in the chart to represent variability among replicates. Pigment production was monitored daily by measuring the absorbance of the culture-filtrate, diluted 1:10 with distilled water, at the pigment’s λ_max_ (350 nm). Based on the Beer–Lambert law, pigment absorbance is directly proportional to its concentration^10^; thus, pigment yield was expressed in terms of absorbance values. Mycelial mats were dried at 60 °C for 24 h after being filtered through Whatman No. 1 filter paper and weighed daily by the benchtop balance (RADWAG-AS 220/C/2) to determine the fungal biomass dry weight (g/L).

### Pigment extraction and purification

The solvent extraction (SE) was used to extract the extracellular water soluble pigment from the fermentation medium according to^11^. For extraction, equal amounts of solvent mixture (n-hexane, ethyl acetate, dichloromethane, and propanol (1:1:1:1)) and the pigment-containing filtrate were vigorously shaken for 5 min in a separating funnel and left for 10 min until clear separate phases were formed. This step was repeated three times using a freshly prepared solvent mixture each time. To remove proteins, the pigment-containing aqueous phase was vigorously mixed with ice-cooled 99% ethanol, causing protein precipitation. Then, the alcohol mixture was centrifuged (10,000 rpm) for 10 min by Centurion Scientific Benchtop Centrifuge (K241R-15002–8). The purified pigment-containing supernatant was collected in a clean vial, and the protein precipitate was discarded. The purified aqueous pigment (PP) was freeze-dried by bench top lyophilizer (VirTis Freeze Dryer sentry 2.0), and the resulting powder was stored at -20 °C for further use^9^.

### Pigment characterization

To test solubility of the pigment powder, some organic and inorganic solvents (methanol, ethanol, petroleum ether, chloroform, acetone, butanol, dichloromethane, n-hexane, and water) and combinations of them were used. For pigment structure elucidation, some phytochemical analyses, (UV–vis.) spectroscopy, HPLC, FTIR, TLC, LCMS and NMR were carried out.

#### Qualitative phytochemical analysis

Phytochemical analysis for the presence of phenols, flavonoids, alkaloids, terpenoids, steroids, sterols, amino acids, proteins, and carbohydrates was performed according to Saravanan et al., (2020) as explained in (Supplementary Table S1).

#### Pigment stability

The pigment stability was evaluated according to Suwannarach, et al.^12^, under pH range 0–14 and temperatures from 30 to 100 °C. The pigment solutions (0.1 mg/mL) were prepared in 99% ethanol and their pHs were adjusted using 1 M NaOH or 1 M HCl and incubated for two h at room temperature. Thermal stability of the pigment solutions was assessed at pH 6.0 (the pigment production pH) by incubating them in a water bath (Azotta-SDK-12) at temperatures between 30 and 100 °C for 5 h. The absorbance at 300 nm (λ_max_) was measured for each condition using 1:10 diluted samples to evaluate color stability. The pigment’s stability percentage was calculated using the formula: $Stability (\% ) = (As/A0) \times 100$, where $"As"$ is the absorbance after treatment and "${\mathrm{A}}0$" is the initial absorbance. In addition, the purified pigment (PP) solution was stored on the shelf at room temperature for six months to check for pigment color change and precipitation. All experiments were conducted in triplicates.

#### Ultraviolet visible (UV–vis.) spectroscopy

The lyophilized pigment was dissolved in 99% ethanol and used to determine its absorbance spectrum by UV–vis spectrophotometer (Helios Alpha, 9423 UVA 1002E spectrophotometer, central lab., Faculty of Pharmacy, Alexandria University, Egypt). The scanning range was 200–600 nm, and ethanol (99%) was used as a blank^13^.

#### High performance liquid chromatography (HPLC) analysis

Twenty microliters of the pigment were injected into Agilent 1100 HPLC equipped with a quaternary pump (P2000), a degasser (G1322A), an autosampler (AS4000), a fraction collector, and SPD-M20A photo diode array detector. For sample preparation, 25 mg of PP were dissolved in 1 mL of deionized water and Acetonitrile (1:1), and the solution was passed through a 0.45 μm membrane filter. The separation was performed using HyperClone ODS C18 Column, Phenomenex (250 × 10 mm-5 μm particle size). The mobile phase consisted of water (0.1% formic acid; eluent A) and acetonitrile (0.1% formic acid; eluent B), using a gradient program as follows: 0 min, 5% B; 15 min, 95% B; 17 min, 95% B; 18 min, 5% B. The flow rate was 0.5 mL/min, the oven temperature was 30 °C, and the capillary voltage was 220 VAC + /- 10%. The chromatogram of UV–vis. spectrum was measured at 300 nm and the quantitative data were processed using ChemStation software version 1997–2003. This method is quite similar to that used by Medic, et al.^14^ who used HPLC with photodiode array detector at 280 nm for quantification of 1,4-naphthoquinone glycosides (Hydrojuglone rhamnoside and Hydrojuglone pentoside), using C18 column and similar mobile phase and gradient system. The collected fractions were lyophilized to powder and stored in the refrigerator for further analyses. Similar HPLC methodology was employed by Dallmann, et al.^15^ for quantification of Lawsone and hydrolawsone which are 1,4-naphthoquinone derivatives.

#### Thin layer chromatography (TLC)

For pigment profiling, the PP and its two fractions were first dissolved in 99% ethanol. Samples were then applied to Silica Gel 60 F254 aluminum TLC plates using a capillary tube and left to air-dry. For pigment separation, a methanol–water mixture (4:1, v/v) served as the mobile phase^16^. To analyze the sugars associated with the pigments, the same TLC plates were run in a different solvent system consisting of ethyl acetate–methanol (6:4, v/v). After each run, the plates were air-dried. Pigment spots were visualized and imaged by UVLS-28 EL SERIES UV LAMP, 6W (95–0201-02), at 365 nm, whereas sugar spots were revealed by spraying the plates with p-anisaldehyde^17^. As a negative control, TLC plates were imaged before p-anisaldehyde staining to confirm the absence of initial carbohydrate-specific spots. Finally, the R_f_ values of the detected spots were calculated using Eq. (1):

1 $${R}_{f}=\text{Distance travelled by sample }/\text{Distance travelled by solvent}$$

#### Fourier transform infra-red spectroscopy (FTIR)

The IR spectra of PP and its two fractions were defined using Bruker FTIR spectrometer (MPA model) in the Central lab. Unit, Faculty of Pharmacy, Alexandria University. This was performed by mixing 5 mg of the sample powder with 200 mg KBr and pressing to form pellets. KBr-pelleted samples were analyzed in the range 4000–400 cm^−1^ at 27 °C with 50 scans and 4 cm^−1^ resolution^18^.

#### LCMS

To determine the molecular mass of the pigment, both fractions were analyzed by Shimadzu LCMS 2020 system equipped with a photodiode array detector and fitted with an Electrospray Ionization (ESI) source, supporting both positive and negative ionization modes. First, Chromatographic separation was performed on a Shim-pack XR-ODS II column (100 × 3.0 mm, 2.2 μm particle size). The mobile phases consisted of water with 0.1% formic acid (eluent A) and acetonitrile with 0.1% formic acid (eluent B), applying the following gradient program: 0 min, 5% B; 15 min, 95% B; 17 min, 95% B; 18 min, 5% B. The flow rate was maintained at 0.2 mL/min with an injection volume of 10 μl. Samples were prepared in a 1:1 mixture of acetonitrile and water, and the column oven was kept at 30 °C. Following, mass detection was performed on the Mass Spectrometer under the following conditions: ESI in both positive ( +) and negative (–) modes, nitrogen as the nebulizing gas at 1.5 L/min, drying gas at 15 L/min, a mass-to-charge ratio (m/z) scan range of 50–800, interface voltage of ± 4.5 kV, desolvation line temperature of 250 °C, and heat block temperature of 200°C^9^.

#### Nuclear magnetic resonance (NMR)

The ^1^H and ^13^C NMR spectra of both pigment fractions were conducted on Jeol NMR spectrometer- 500 MHz (JNM-ECZ500R/S1). ^1^H and ^13^C NMR spectra were recorded using DMSO-d6 as solvent^19^. The samples were prepared at a concentration of 10 mg/mL, and results were recorded in 6 mm NMR tubes applying standard ^1^H and ^13^C pulse programs. In case of ^13^C, field Strength was 11.7473579 Tesla, Carbon-^13^ frequency was 125.76529768 MHz, and experimental parameters: pulse sequence scans, relaxation delay, acquisition time, and temperature were single pulse decoupling, 2042, 2 s, 0.82837504 s, and 15.4 °C, respectively^20^. The proposed pigment structure was interpreted from the characterization data and drawn using Chemoffice 2015 software.

## Results

### Pigment production

The pigment-producing fungal strain was isolated and identified as *Aspergillus unguis* AUMC 15,225 (supplementary figure S1, a) based on morphological characteristics (supplementary figure S1, b) and ITS rDNA sequence analysis. The ITS sequence was deposited in GenBank under accession number OR398781, and a voucher specimen is maintained at the Assiut University Mycological Center (AUMC). The fungus was used to inoculate the fermentation medium which was daily monitored for visible color changes. After nine days, the medium began to turn red, and the color deepened gradually. The UV–visible absorption spectrum of the pigment-containing filtrate (Supplementary Figure S2) showed a characteristic peak, with λ_max_ of 350 nm before pigment extraction and purification.

### Growth and pigment production kinetics

**Fig. 1:**
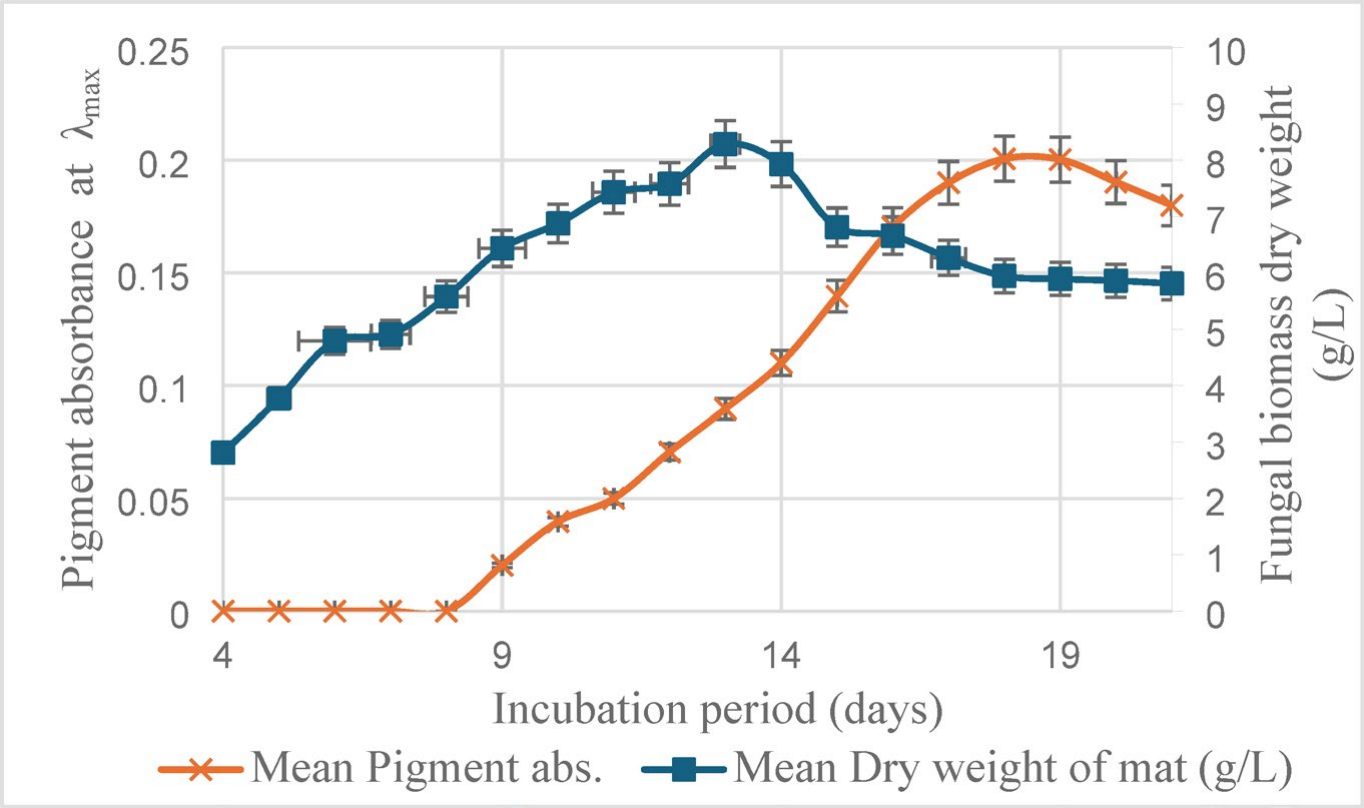
Kinetics of fungal growth and pigment production by *A. unguis* AUMC15225.

The progress of growth and pigment secretion was monitored throughout the fermentation period **(**Fig. 1**)**. Pigment absorbance started to rise by day nine and reached its maximum (0.20) by day 1, then started to decline. Biomass dry weight started to increase from day four (2.813 g/L) and peaked on day 13 to 8.28 g/L, then gradually declined, reaching 5.81 g/L at the end of incubation.

### Pigment extraction and purification

**Fig. 2:**
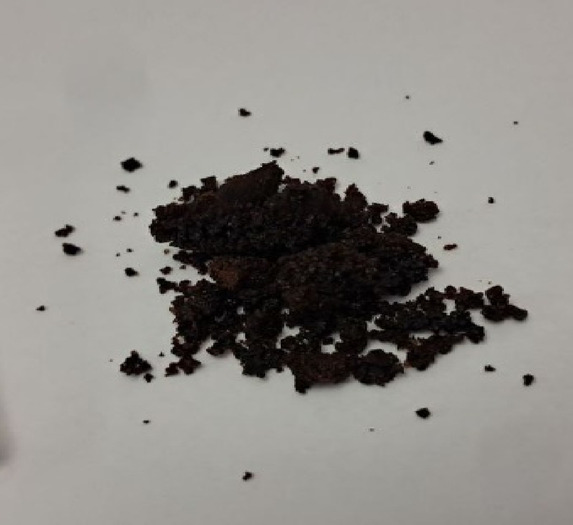
The lyophilized pigment powder.

During extraction, all nonpolar solvents couldn’t extract the pigment that remained in the aqueous phase, while the non-polar layer changed from colorless to faint yellow. By the third extraction cycle, no further color change was observed in the non-polar phase. After protein precipitation and filtration, a purified aqueous pigment fraction was obtained and lyophilized into a dark reddish-brown powder (Fig. 2).

### Pigment characterization

Solubility testing has revealed that the pigment powder was completely soluble in 99% ethanol. Then, the phytochemical analyses and stability assessments were conducted on the PP that was further exposed to HPLC fractionation using a C18 column. The two collected fractions were characterized using various chemical techniques, including UV–vis. Spectroscopy, TLC, FTIR, LCMS, and NMR to elucidate the pigment’s chemical structure.

#### Phytochemical analysis

The analyses confirmed the absence of phenols, flavonoids, steroids, sterols, amino acids, and proteins. Wagner’s test yielded a reddish-brown precipitate, confirming the presence of alkaloids (Supplementary Figure S3, a). Moreover, the Salkowski test produced a reddish-brown-colored layer at the mixture top, indicating the presence of terpenoids (Supplementary Figure S3, b). Additionally, the Molisch test resulted in the formation of a violet ring in the interface, confirming the presence of carbohydrates (Supplementary Figure S3, c).

#### Pigment stability

The pigment demonstrated high stability under the tested conditions. It showed higher resilience to temperature fluctuations than to pH changes, with the highest stability (100%) recorded at pH 6 and at 30–40 °C (Supplementary Figure S4). The lowest stability recorded was 94.5%, at pH values of 0, 12, and 14, and 96.4% at 80 -100 °C. Moreover, no precipitation or color change was detected during six months of shelf storage.

#### UV–visible spectroscopy

**Fig. 3:**
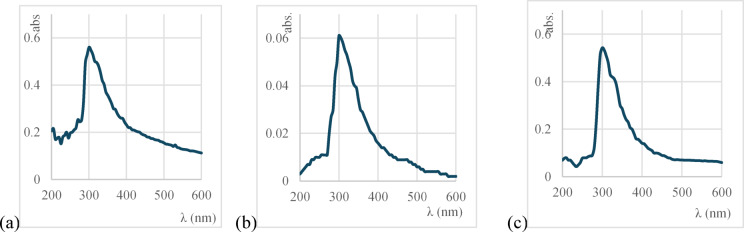
UV–visible spectrum (**a**) PP, (**b**) fraction 1 and (**c**) fraction 2.

The UV–visible spectra of PP and its fractions showed that all of them are sharing the same λ_max_ of 300 nm (Fig. 3).

#### HPLC and fraction collection

**Fig. 4:**
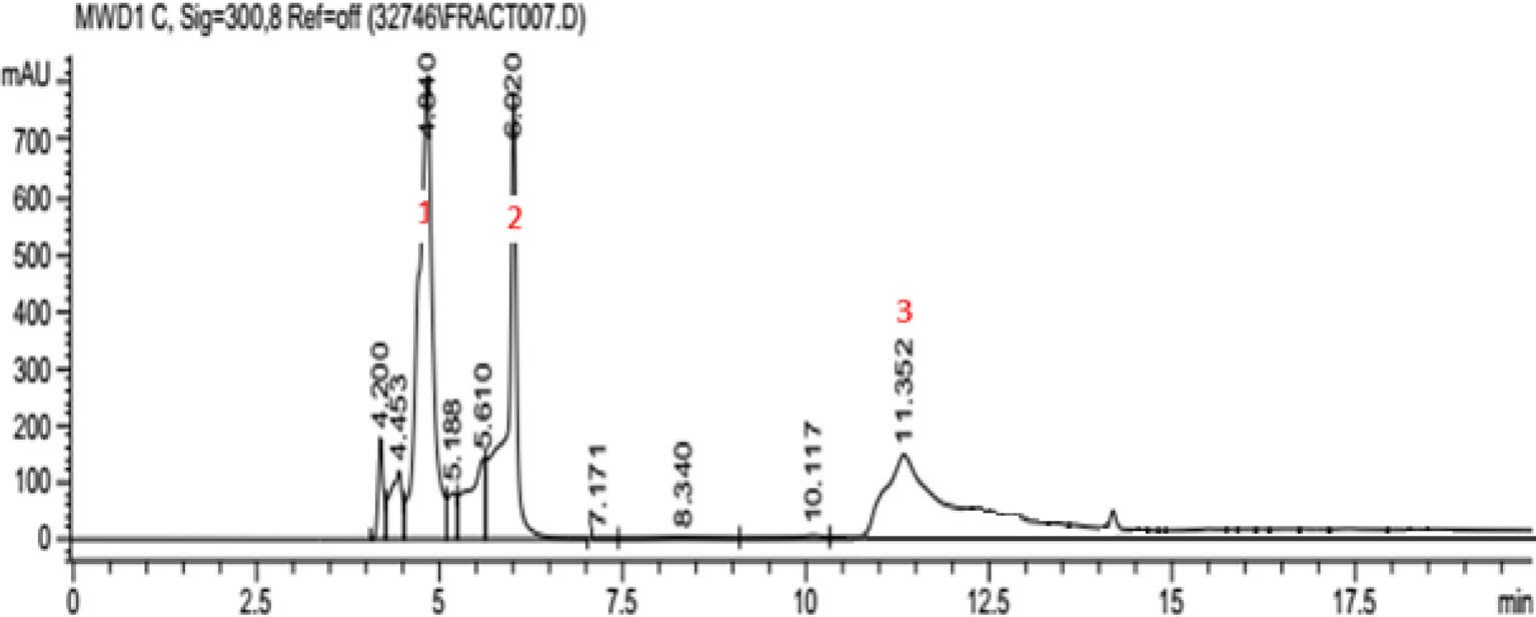
HPLC analysis of the pigment using HyperClone ODS C_18_ Column.

The pigment HPLC chromatogram (Fig. 4) showed three major peaks (RTs 4.840, 6.020 and 11.352). Only fractions 1 and 2, that displayed the pigment’s color, were retained and dried for further characterization, while fraction 3 was colorless and excluded.

#### TLC

The R_f_ values were 0.8, 0.76 and 0.8 for PP, first and second fractions respectively. The R_f_ of the attached carbohydrate (green) spot at 0.47 was detected only after p-anisaldehyde spraying, while no spot was observed on the plate prior to spraying, confirming the specificity of sugar detection (Supplementary Fig. S5).

#### FTIR

**Fig. 5:**
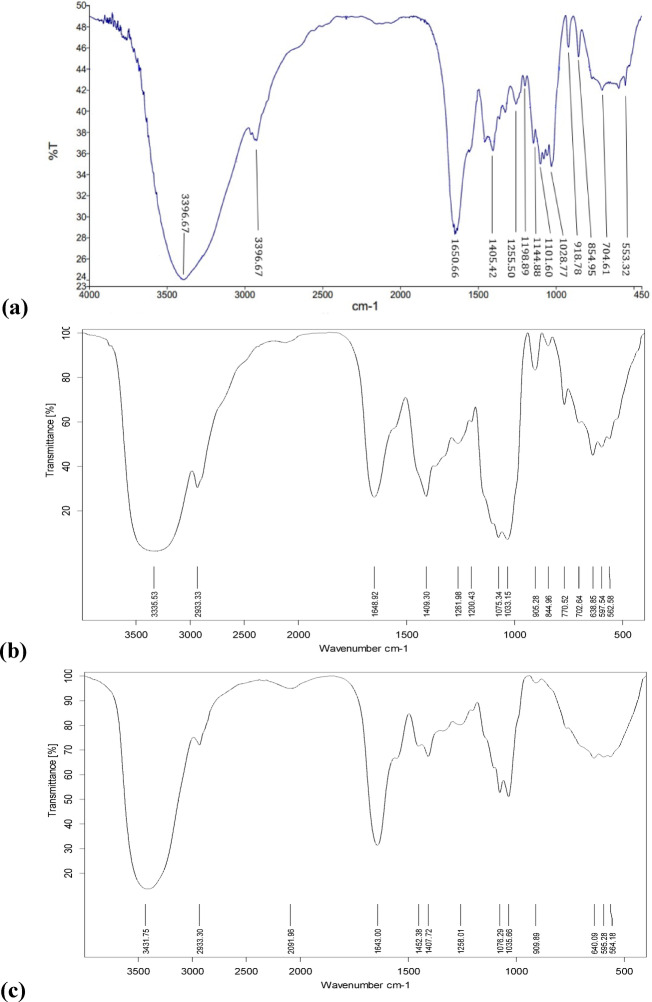
FTIR spectra of (**a**) PP, (**b**) Frac. 1 and (**c**) Frac. 2

For better understanding of the chemical structure, FTIR was conducted to determine the main functional groups^21^ of the pigment and its fractions (Fig. 5). Three common peaks were observed in the three spectra. The first was at 2926.30 cm^-1^ in PP and at 2933 cm^1^ in both frac. 1 and 2. The second was at 1405.42 cm^-1^ in PP, 1409.30 cm^-1^ in frac. 1 and 1407.72 cm^-1^ in frac. 2. The third was at 1255 cm^-1^ in PP, 1261.97 cm^-1^ in Frac. 1 and 1258.00 cm^-1^ in Frac. 2. Moreover, PP and fraction 1 share three common peaks. The first was 1650 cm^-1^ in PP and 1648 cm^-1^ in frac. 1. The second was 1198.89 cm^-1^ in PP and 1200.42 cm^-1^ in frac.1. The third was 704 cm^-1^ in PP and 702 cm^-1^ in frac. 1. On the other hand, 7 peaks were unique to PP spectrum at; 3396 cm^-1^, 1144 cm^-1^, 1101 cm^-1^, 1028 cm^-1^, 918 cm^-1^, 854 cm^-1^ and 553 cm^-1^.

#### LCMS

**Fig. 6:**
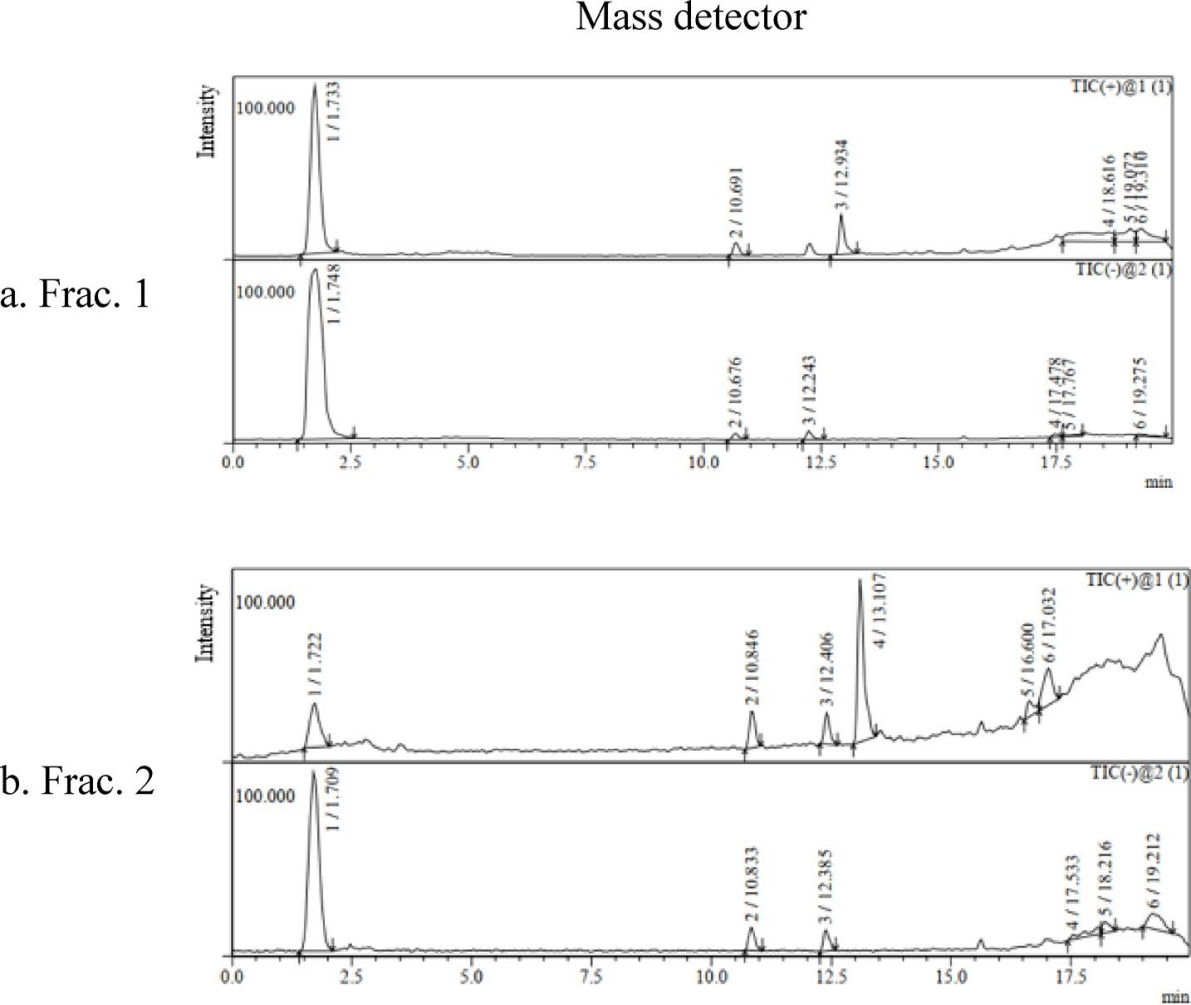
LCMS spectra by mass detector for Pigment fractions; (**a**) 1 and (**b**) 2.

LCMS screening via Electrospray Ionization Mass Spectrometer has revealed that both fractions’ spectra (Fig. 6) were highly similar in TIC positive and negative modes. In frac. 1, four major peaks were detected at RTs of 1.733, 10.691, 12.243 and 12.934 min in TIC ( +) and four major peaks at 1.748, 10.676, 12.243 and 17.478 min in TIC (-). Also, frac. 2 showed four major peaks at RTs: 1.722, 10.846, 12.406 and 13.107 min in TIC ( +) and four peaks at RTs: 1.709, 10.833, 12.385 and 17.533 min in TIC (-). Mass fragmentation’ spectra of all peaks at each RT are available in Supplementary Table S2.

#### NMR

**Fig. 7:**
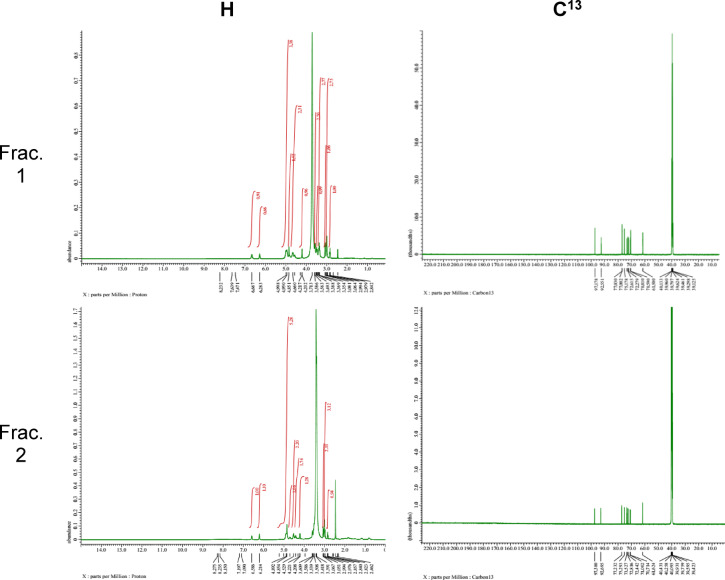
^1^H and ^13^C NMR spectra of (**a**) frac. 1 and (**b**) frac. 2

NMR spectrometry was used for deeper understanding of the pigment chemical structure and the ^1^H spectrum (Fig. 7) of both pigment fractions were highly similar and revealed many significant chemical shifts. In the aromatic region (6.0—9.0 ppm); frac. 1 showed two peaks at 6.667 and 6.283 ppm and fraction 2 showed two peaks at 6.586, 6.214 ppm. In the region around 4–5 ppm; fraction 1 showed peaks at 4.998, 4.950, 4,851, 4.660, 4.217 ppm, while fraction 2 showed peaks at 4.892, 4.854, 4.529, 4.221 ppm. In the aliphatic region (3–4 ppm), fraction 1 showed peaks at 3.713, 3.586, 3.493 and 3.388 ppm, while fraction 2 showed a peak at 3.418 ppm. Also, fraction 1 showed a peak at 3.081 ppm and two at 3.051 ppm. In the region 2–3 ppm, frac. 1 showed peaks at 2.994, 2.842 and 2.4 ppm and fraction 2 showed similar peaks at 2.994, 2.840 and 2.462 ppm.

**Fig. 8:**
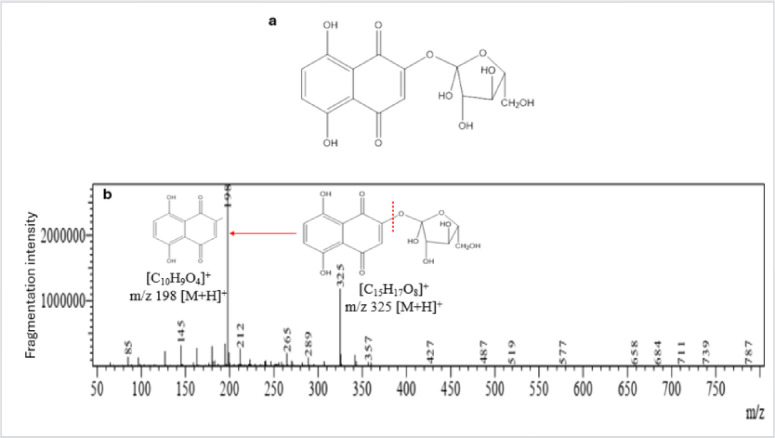
Proposed chemical structure and MS fragmentation pathway of the pigment. (**a**) Chemical structure of 2-O-β-L-arabinofuranosyl-5,8-dihydroxy-1,4-naphthoquinone. (**b**) Proposed fragmentation pattern in positive ionization mode (TIC +), illustrating the loss of the arabinose sugar moiety and subsequent fragmentation of the naphthazarin core ([C_10_H_9_O_4_]^+^).

In case of ^13^C NMR, both fractions revealed high similarity and fraction one showed the following significant peaks; 97.1, 92.5, 77.0, 75.1, 72.6, 72.2, 70.8, 70.5, 61.5, 40.1, 39.9, 39.7, 39.6, 39.4, 39.2 and 39.1 ppm. While fraction showed similar significant peaks as follows; 97.3, 95.77, 92.6, 77.2, 75.2, 73.5, 72.8, 72.4, 71.0, 70.7, 61.6, 40.4, 40.2, 40.0, 39.9, 39.7, 39.5 and 39.4 ppm (Fig. 6). The molecular formula of the pigment is expected to be C₁₅H₁₆O₈, and its proposed chemical structure and TIC + mass fragmentation is shown in Fig. 8.

## Discussion

In the present study we used the epiphytic mangrove fungus, *A. unguis* to produce the extracellular, water soluble, arabinofuranosyl naphthazarin pigment. During the fermentation, the pigment changed the medium’s color indicating the pigment extracellular and water-soluble nature. The pigment-containing filtrate exhibited a λ_max_ at 350 nm, which falls within the typical range of naphthoquinone derivatives (300–360 nm)^22^. This absorption is characteristic of the quinone core transitions near 300 nm and could extend to around 280–360 nm, depending on conjugation or substituent effects in NQ derivatives^23^.

The pigment yield has peaked after biomass began to decline, indicating its secondary metabolic behavior. This is typical of fungal secondary metabolites, that are usually produced when growth declines shifting from biomass accumulation to stress adaptation due to nutrients depletion and unfavorable conditions. Those metabolites often provide survival advantages such as protection against UV radiation, microbial competition or oxidative stress^24^.

The extracellular water-soluble pigment extraction is less complicated than intracellular pigments, which requires preliminary biomass disruption and separation^25^. We applied solvent extraction method which is very efficient in recovery of extracellular pigments from plants and fungi^11^. During extraction, the pigment remained in the aqueous phase and was not recovered with the nonpolar solvents, indicating its high polarity. This agrees with previous articles reported the high polarity of glycosylated naphthoquinones^7^. Similarly, Dulo, et al.^6^ had reported that solvent mixtures with alcohol and water increase the yield of glycosidic naphthoquinones. In this study, the nonpolar solvent phase shifted from colorless to yellow, likely due to the removal of less polar metabolites from the culture filtrate. After three successive extractions, no further color change in the nonpolar phase was observed, indicating complete removal of these impurities. Solubility testing further supported the pigment’s high polarity that dissolved only in 99% ethanol or lower concentrations, consistent with previous reports that ethanol is an efficient solvent for naphthoquinones^6^.

Chemical characterization of microbial pigments is essential to understand their structure, properties and the structure–function relationship^26^. Phytochemical analyses confirmed absence of phenols, flavonoids, steroids, sterols, amino acids and proteins ensuring the high quality of the performed extraction and purification steps. On the other hand, those analyses confirmed the presence of some kinds of carbohydrates that could be attributed to the pigment-production pathways involving enzymatic glycosylation. Carbohydrate motifs attached to the pigment can enhance solubility, drug delivery and bioactivity^27^. Also, glycosylated NQs are reported to have significant anti-inflammatory, antiviral, antioxidant, antitumor, anticoagulant, and hypolipidemic activities^5,28^.

Pigment stability is defined as resistance to degradation and color change after prolonged exposure to light, high temperature, oxygen, or extreme pHs, which is a critical feature in colorants and paints. In the current work, the pigment overall stability is promising compared to some natural pigments suggesting its potential for a wide range of industrial applications. This is consistent with the reported high thermal stability of NQ derivatives like naphthazarin-calcite^29^. Also, it retained its color across the entire pH spectrum and from 10 to 100 °C. In contrast, the red pigment produced by *Nigrospora aurantiaca*^12^ exhibited color shifting to bluish-red at pH 8.0 and 9.0, purplish-red at pH 10.0, and reddish-violet at pH 11.0. Also, their pigment color stability was high only between 20 to 50 °C and reduced from 50 to 80 °C. Glycosylation is expected to enhance stability through increased water solubility and steric protection of the naphthoquinone core. As evidenced by Aminin and Polonik^30^, the glycosylation of shikonin has significantly improved stability and solubility compared to the parent aglycone. In addition, glycosylation increases hydrophilicity making the molecule more soluble in aqueous environments and prevents aggregation.

The pigment was screened via HPLC and its two fractions were detected at RTs 4.8 and 6 min at 300 nm which is very close to lawsone and hydrolawsone^15^ and Juglone^31^. Furthermore, the pigment and its fractions shared the same λ_max_ 300 nm, indicating common naphthoquinone core. This is within the reported range of 1,4-naphthoquinone derivatives’ λ_max_ at 280–368 nm depending on the attached substituents, the used solvents^23^ and their concentrations^6^. It’s also close to that of fusarubin (1,4-napthoquinone derivative) produced by *Fusarium chlamydosporum* at 316 nm^32^.

The TLC R_f_ value of the pigment (0.76–0.8) falls within the range reported for naphthoquinone pigments isolated from the fungal species *Fusarium moniliforme* MTCC 6985 (Rf 0.6–0.9), providing a biologically relevant qualitative comparison^33^. In addition a structurally related NQ derivative ( 5,8-dihydroxy-1,4-naphthoquinone thioglucoside) has been reported to exhibit a polar TLC band with an Rf value of approximately 0.86^34^, which is consistent with the chromatographic behavior observed in the present study. Moreover, the R_f_ of the carbohydrate attached to the pigment (0.48) is very close to that of arabinose sugar (0.47) produced by *Rhodospirillum* sp.^17^. This supports the polarity and chromatographic behavior rather than definitive identification.

FTIR was recruited for deeper insight into the functional groups of the pigment and its fractions. The high degree of resemblance in their IR spectra supports the suggested structural similarity. While the three spectra shared three common peaks, the first peak at 2920–2933 cm⁻^1^ was reported as a characteristic peak in a thioglucoside derivative of 1,4-naphthoquinone^35^. It was also reported to be corresponding to a strong intramolecular hydrogen bonding between the α hydroxyls and the quinonoid carbonyls in naphthazarin pigment (5,8-dihydroxy-1,4-naphthoquinone)^36^. The second peak at 1405–1409 cm⁻^1^ might be related to C-O–H bending or CH_3_ asymmetric deformation^37^. The third peak at 1255–1262 cm⁻^1^ is due to characteristic vibrations of aromatic group^38^ and was reported as a characteristic peak in a thioglucoside derivative of 1,4-naphthoquinone^35^. Presence of peak 3396 cm^-1^ in PP, 3335 cm^-1^ in fraction 1 and 3431 cm^-1^ in fraction 2 is related to β hydroxyl groups of naphthazarins indicating that β isomer is the dominant^35^. Then, PP and fraction 1 shared three additional peaks. The 1650 cm⁻^1^ peak is characteristic for quinonic carbonyl groups of NQs^39^. The 1198–1200 cm⁻^1^ peak are attributed to C-O stretching in glycosylated derivatives of 1,4-naphthoquinone^35^. The peak around 700 cm⁻^1^ might be due to out-of-plane bending of aromatic C-H, common in substituted benzene rings of NQs^40^. The unique peaks in PP like 1144, 1101, 1028 cm⁻^1^, could be due to C–O–C or glycosidic linkages. The peaks at 918, 854 and 553 cm⁻^1^ might be due to C-H deformation of benzene ring^41^, C-O bending of carbonate^42^ and C_α_ = C_α΄_ torsion or ring torsion^43^, respectively. The pigment’s glycosylation pattern was evident from the peaks at 3396, 1200–1260, and 1028 cm⁻^1^, suggesting that the attached glycone is likely an arabinose unit^43,44^.

LCMS was employed for pigment profiling and confirmed the isomerism of both fractions. They showed highly similar elution pattern with matching retention times at ~ 1.7, 10.6–10.8, and 12.2–13.1 min in both ionization modes. Their similar peak intensities reflect comparable concentrations and fragmentation patterns were also high similarity. For example, both fractions showed a peak at ~ 1.7 min that fragmented into the same parent ions (m/z 325 in TIC + ; 255 in TIC-) and base ions (m/z 198 in TIC +). This fragmentation pattern in TIC + suggests a cleavage of the glycosidic bond releasing a fraction of pentose sugar (arabinose) of 126 Da (324—198), and an ionized aglycone (5,8-dihydroxy-1,4-naphthoquinone core) of 255 Da. It’s common for isomers with the same molecular weight to have similar elution patterns and very close RTs and peak intensities under similar analysis conditions^45^. Their similar fragmentation strongly supports the idea that both fractions have the same molecular structure^46^. HPLC separation of both isomers into two distinct fractions and the little variation in their fragmented ions masses (m/z) indicated the difference in polarity because of varied stereochemical configuration^47^. The peak at RT of 1.7 in TIC + and TIC- is the most intense and represents the most dominant protonated molecule (m/z 325)^48^ with a molecular weight of 324 Da for the neutral molecule. TIC- did not show the expected deprotonated ion [M-H]⁻ at m/z 323 but a parent ion at m/z 255. This suggests the compound fragmentation during negative ionization and losing a neutral fragment of 69 Da via the detachment of a hydrocarbon chain (C₅H₁₀) from the aglycon. This demonstrated the high stability of the protonated molecule [M + H]⁺ in TIC + compared to TIC- mode. This is consistent with Flores, et al.^49^ who reported that some compounds can undergo neutral losses like decarboxylation more readily in one ionization mode. This could be attributed to the lack of strong electron-withdrawing groups that stabilize the deprotonated form. This ionization behavior confirms the pigment high polarity explaining early elution in reversed-phase LC, as polar compounds interact less with the hydrophobic stationary phase^50^. Additionally, the protonated ion (m/z 325) produced a pentose neutral loss of 132 Da (C₅H₈O₄) ion, yielding m/z ≈ 193, which corresponds to the protonated aglycone (C₁₀H₈O₄ + H). Subsequent fragments consistent with loss of CO (28 Da) and CO₂ (44 Da) from the aglycone were also observed, supporting the naphthoquinone core assignment. Accordingly, the pigment protonated molecule [M + H]^+^ at m/z 325.09 corresponds to the molecular formula C_15_H_16_O_8_ (calculated 325.0918 Da). This formula represents a pentose-glycosylated naphthoquinone, consisting of a naphthazarin aglycone (C_10_H_6_O_4_) and an arabinose unit (C_5_H_10_O_5_) following the loss of one water molecule during glycosidic bond formation (C_10_H_6_O_4_ + C_5_H_10_O_5_—H_2_O = C_15_H_16_O_8_) and was consistent with the proposed structure: 2-O-β-L-arabinofuranosyl-5,8-dihydroxy-1,4-naphthoquinone (Fig. 7).

The high similarity in mass fragmentation patterns, shared λ_max_, and highly similar FTIR and NMR spectra of both fraction 1 and fraction 2 strongly suggest that they are stereoisomers (diastereomers) of the proposed compound, 2-O-β-Larabinofuranosyl-5,8-dihydroxy-1,4-naphthoquinone. The separation into two distinct fractions by HPLC (Fig. 3) confirms they are not simple conformers. As they share structural core, the difference is almost due to epimerism at a chiral center of the sugar moiety. Enzymatic (e.g., epimerases) interconversion between both is biologically possible but would require specific enzymatic activity^51^, which is not confirmed in this study. Moreover, chemical interconversion (epimerization or mutarotation) is generally slow for glycosidic linkages under mild conditions ^52^. While the structural similarity of both isomers is obvious, their absolute stereochemistry (e.g., the precise configuration at the furanose ring carbons) was not determined and represents a limitation of the current study.

The ^1^H NMR spectrum (500.16 MHz, DMSO-d₆) revealed characteristic signals of the 5,8-dihydroxy-1,4-naphthoquinone (naphthazarin) core, with two broad aromatic singlets at δ 6.586 and 6.214 ppm, assigned to H-6 and H-3, respectively^36,53,54^. The β-stereochemistry of the glycosidic linkage is confirmed by a doublet at δ 4.892 ppm, that was identified as the anomeric proton (H-1′) of the glycosidic sugar^54,55^, with coupling constant of 8 Hz, which is characteristic of a β-configured glycosidic linkage in furanosides^56^. Also signals between δ 4.85–4.22 correspond to the remaining protons of the pentose sugar unit^54,57^. Also, the peaks at 2–3 ppm correspond to the DMSO signal^58^. The 13C NMR spectrum showed a characteristic signal of anomeric carbon, C-1′ at δ 97.38 and signals of oxygenated sugar carbons at δ 77–70 ppm^59^ of the pentose sugar. In addition, the signal at δ 61.62 ppm is attributed to a hydroxymethyl carbon (C-5′) that confirms the sugar is in furanose form with a free CH_2_OH group^60^. Moreover, a signal at δ 40.4 ppm was assigned to the C-2′ carbon of the naphthoquinone aglycone which is a spectroscopic signature for O-glycosylation^61^. The naphthazarin core is known to exist in tautomeric equilibrium between quinone and enolized forms in case of glycosylated systems or using polar solvents such as DMSO-d₆. In the molecule of this study, the two free hydroxyl groups (5,8-OH) are expected to be engaged in intramolecular H-bonding (C = O ↔ C–OH). Such intramolecular hydrogen bonding can lead to broadened or shifted carbonyl carbon resonances in the ^13^C NMR spectrum, making its signal assignment complicated,^62^ explaining its absence in the current spectrum. This is also confirmed by the FTIR peaks at 2920–2933 cm^-1^ that represent intramolecular hydrogen bonding between the quinonoid carbonyls and α hydroxyls the in naphthazarin core^36^. Peak clusters around 39–40 ppm may be caused by the DMSO signal^58^. Collectively, NMR results in combination with other characterization analyses provide structural verification confirming the proposed structure (2-O-β-L-arabinofuranosyl-5,8-dihydroxy-1,4-naphthoquinone).

Research on glycosylated naphthazarins from natural sources remains relatively scarce, with most studies focusing on the synthetic glycosylation. While the derivative in this study possesses a β-L-arabinofuranose unit attached via a β-glycosidic linkage at the O-2 position of the naphthoquinone core, many synthetic analogues have D-glucopyranose linked at the same or different positions. The synthetic NQ reported by Sabutski, et al.^63^ has a tetra-O-acetyl-β-D-glucose sugar attached at the same C-2 phenolic –OH with polymethoxy-substituted core. In addition, glycosylated Shikonins a distinct subclass where α-D-glucopyranose glycosylation occurs via an alkyl side chain at C-2, rather than direct attachment to the quinone oxygen^64,65^. In addition, mono-glycosylated naphthopurpurin has a D-glucopyranose moiety attached at C-2 with β-1-O-glycosidic linkage^66^. More complex, multi-glycosylated NQ such as acetylated tris-O-glucoside echinochrome and glycosylated spinazarine, have D-Glucopyranose attached via β-1-O-glycosidic linkages at positions (C-2, C-3 and C-7) and (C2 and C3), respectively, resulting in higher molecular weights^66^. On the other hand, the compound in the current study has a pentose (β-L-arabinofuranose) sugar linked via an O-glycosidic bond to the naphthazarin core. Its natural origin, in contrast to synthetic derivatives, supports its unique structure among naphthazarin glycosides.

## Conclusion

This work has recruited the epiphytic mangrove fungus *A. unguis* AUMC15225 to produce a novel glycosylated derivative of naphthazarin extracellular pigments. The growth-phase dependent pattern of pigment production confirmed its secondary metabolic nature since its concentration peaked only at the decline phase. The pigment’s high polarity was demonstrated by its solubility profile, extraction behavior, and early elution during reversed-phase HPLC. Remarkably, the pigment demonstrated exceptional stability, maintaining its color across a broad pH range 0–14 and at temperatures up to 100 °C. Using HPLC, the pigment was separated into two distinct stereoisomers, and their isomerism was confirmed by the same λ_max_, and high similarity of their R_f_ values, FTIR and NMR spectra and LCMS elution patterns. While the primary focus of this work is the core molecular structure elucidation, defining the exact configuration of each furanose ring’s chiral center is a recommended future work. A combination of analytical techniques identified the purified pigment as 2-O-β-L-arabinofuranosyl-5,8-dihydroxy-1,4-naphthoquinone. The presence of a naphthazarin core that is substituted with a an arabinofuranose sugar moiety via a β-glycosidic linkage was supported by the characteristic neutral loss of 132 Da during mass fragmentation and diagnostic signals in the FTIR and NMR spectra. This unique structural composition suggests a specialized biosynthetic pathway that inspires future deeper investigations.

## Supplementary Information

Below is the link to the electronic supplementary material.

Supplementary Material 1

## Data Availability

The datasets supporting the conclusions of this article are included within the article and its supplementary file.
