# Supplementary material for: Extraction and characterization of a novel glycosylated naphthazarin pigment from mangrove Aspergillus unguis AUMC15225
Source: Sci Rep. 2026 Apr 1;16:11238. doi: 10.1038/s41598-026-43500-0 (PMC13046854; doi:10.1038/s41598-026-43500-0)
Supplement: Supplementary file 1 — Supplementary Material 1 [file 41598_2026_43500_MOESM1_ESM.pdf]

Extraction and characterization of a novel glycosylated naphthazarin pigment from Mangrove *Aspergillus unguis* AUMC15225

Basma M. Alkersh[1,2*] · Hanan A. Ghozlan[2] · Soraya A. Sabry[2] · Sahar W.M. Hassan[1] · Amany El-Sikaily[1]
*Correspondence: Basma Mohamad Alkersh, basma.elkersh_pg@alexu.edu.eg
[1]National Institute of Oceanography and Fisheries (NIOF), Alexandria, Egypt.
[2]Faculty of Science, Alexandria University, Alexandria, Egypt.

Supplementary materials

Table S1 Qualitative Phytochemical Analysis

Test	Method	Result	Reference
Phenols test	Tested using ferric chloride test. Forty microliters of 1% $Fecl_3$ were added to 2 ml of pigment aqueous solution. If the pigment contains phenolic fractions, a bluish-black precipitate is going to be formed.	Negative	Saravanan, A. *et al. 2020*[1]
Flavonoids test	Alkaline reagent test was used to check for presence of flavonoids. Few drops of 1% of 1N NaOH were added to 2 mL of the sample in a test tube and left until yellow precipitate is formed. After that, few drops of HCl were added and left for change or disappearance of the yellow color.	Negative	Devi, P., *et al.* 2015 [2]
Alkaloids test	Wagner's test was recruited to check for presence of alkaloids. To prepare Wagner's reagent, 2 g of iodide and 6 g of KI were dissolved in 100 mL of distilled water. Few drops of Wagner's reagent were added at the side of the tube containing 2 ml of the sample and left for the formation of the reddish-brown precipitate	Positive	Saravanan, A. *et al.* 2020 [1]
Terpenoids test	Salkowski test was used to check for presence of terpenoids. 4 mL of chloroform was added to 2 ml of the sample, then 5 mL of H_2SO_4 were added. Next, the mixture was left for the formation of reddish-brown colored layer at the mixture top	Positive	Savithramma, N. *et al.* 2011[3]
Steroids test	Liebermann Burchard Test was employed to test for presence of steroids. 2 ml of chloroform was added to 1 ml of the sample in a test tube. Then, 3 mL of concentrated H_2SO_4 was added at the tube side. The mixture was left to stand until red color formation at the top and changing color of the lower sulfuric acid layer into yellow with green fluorescence	Negative	Bansode, T. S. *et al* 2015[4]
Sterols test	ence of sterols in the pigment was tested using Salkowski test. A mixture of roform and sample (1:1) was mixed in a test tube and the mixture was filtered. filtrate was collected, then, few drops of concentrated H_2SO_4 were added at sides of the test tube. The tube was left until formation of green–yellow color	Negative	Saravanan, A. *et al. 2020*[1]
Amino acids test	Presence of amino acids was tested using the Ninhydrin test. To prepare Ninhydrin reagent, dissolve 10 mg of Ninhydrin in 200 mL of acetone. 2 mL of the sample was mixed with 2 drops of Ninhydrin reagent and left until appearance of purple color	Negative	
Proteins test	Protein presence was checked using Biuret reagent. 2 drops of 2% copper sulfate solution, 2 mL of 70% Ethyl alcohol and KOH pellets were mixed with 2 mL of sample. The mixture was left for formation of pink ethanolic layer	Negative	
Carbohydrates Test	Molisch test was used to check for presence of carbohydrates. To prepare Molisch reagent, α- naphthol was dissolved in 95% ethanol. Two drops of Molisch reagent were mixed with 2 mL of the sample. Then, 2 ml of concentrated H_2SO_4 was added at the sides of the tube. The mixture was left until the appearance of violet ring	Positive	

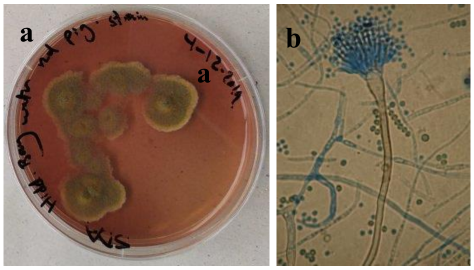

Figure S1. (a) Colonies of *Aspergillus unguis* AUMC15225 on SDA media showing the diffusion of the extracellular pigment into the surrounding agar, (b) A microscopic image of *Aspergillus unguis* AUMC15225.

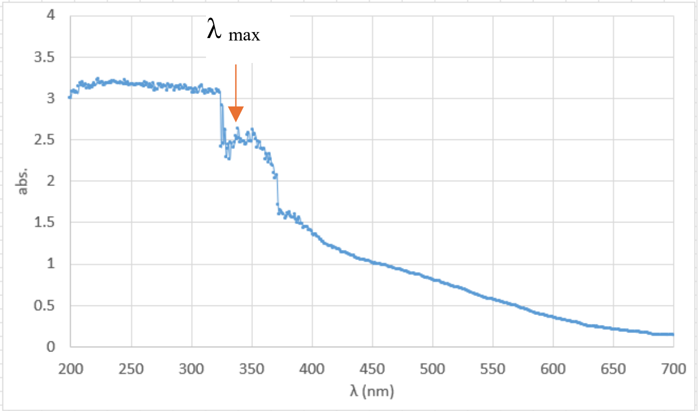

Figure S2. Absorbance spectrum of the pigment-containing filtrate before extraction and purification

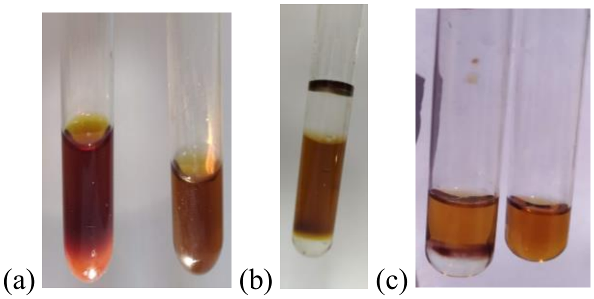

Figure S3. Phytochemical analysis (a) Wanger's test; (b) Salkowski test and (c) Molisch test (control is on the right)

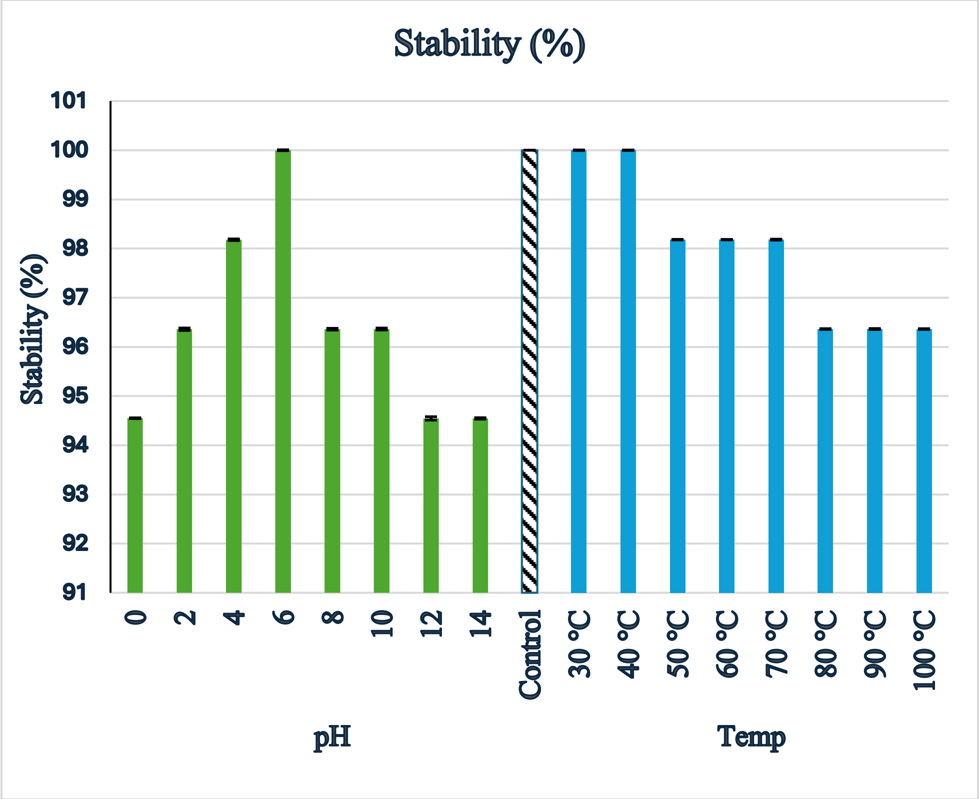

Figure S4. Pigments stability at pHs 0-14 and temperatures 30-100 °C

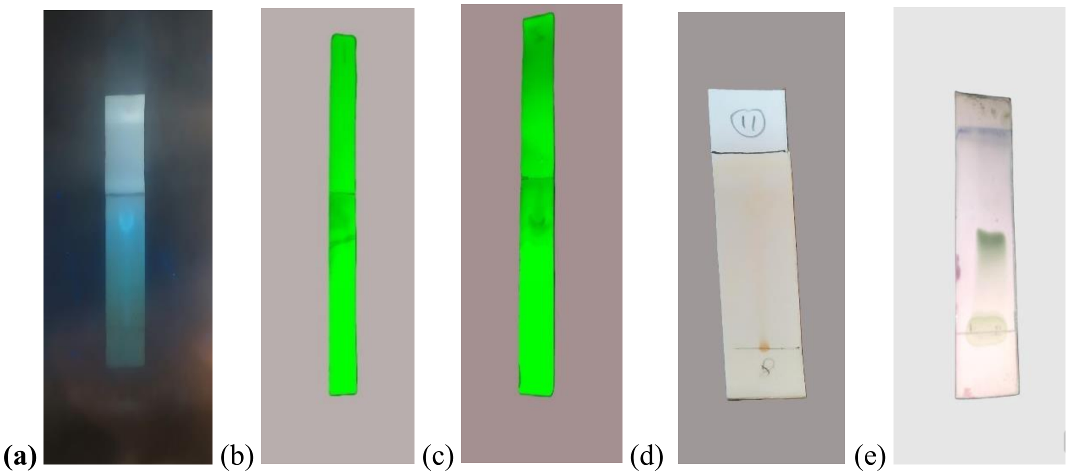

Figure S5. TLC of (a) PP, (b) frac. 1, (c) frac. 2. Plate (d) shows the TLC plate prior to p-anisaldehyde spraying (negative control), while plate (e) shows the TLC plate after p-anisaldehyde staining, revealing carbohydrate-positive spots.

Table S2 Fragmentation chromatograms of fractions 1 and 2

	RT	Frac. 1	Frac. 2
TIC(+)	1.7	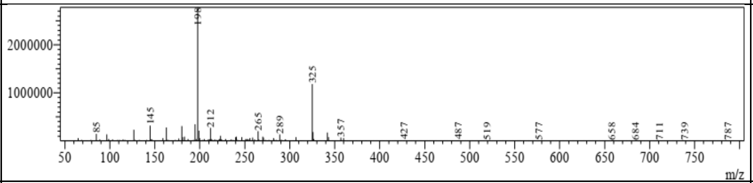	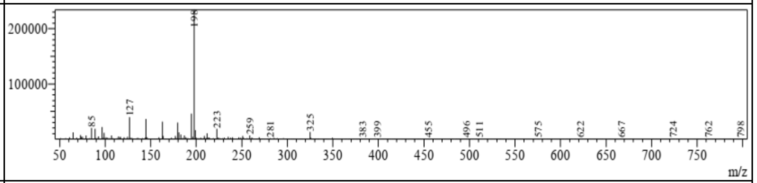
	10.7	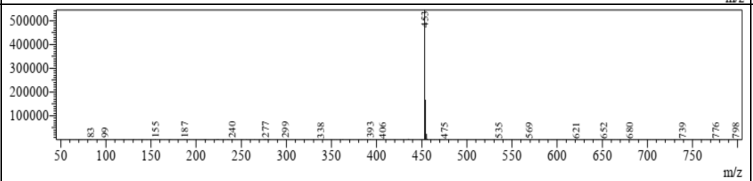	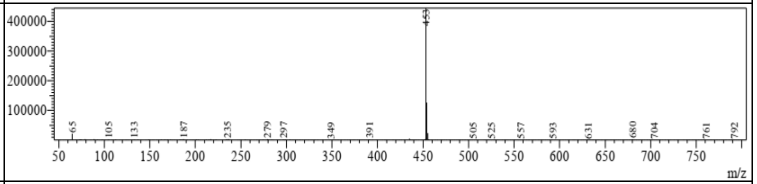
TIC(-)	1.7	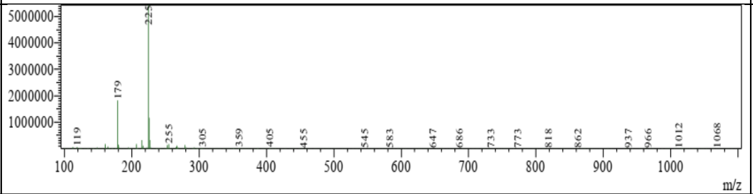	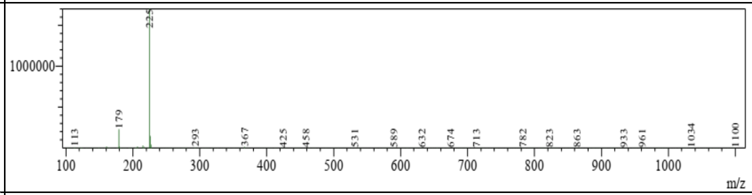
	10.7	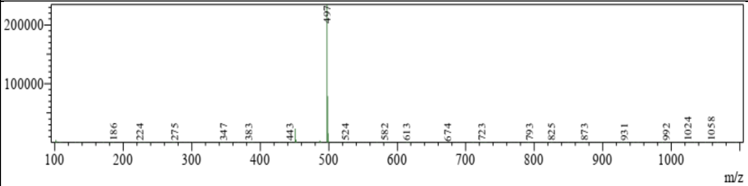	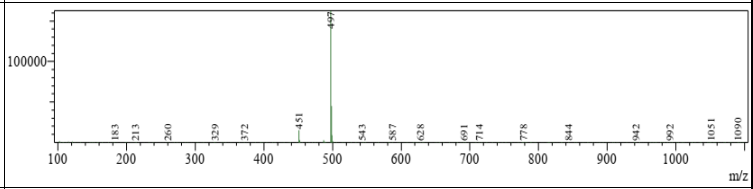
	12.3	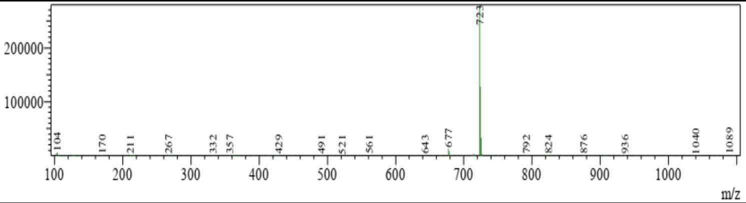	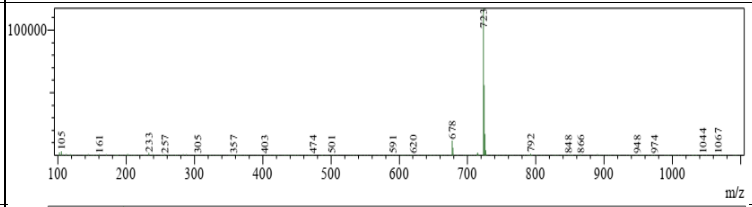
	17.5	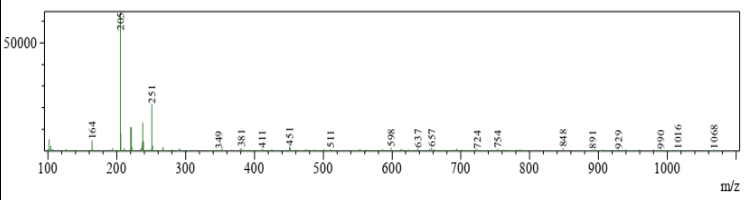	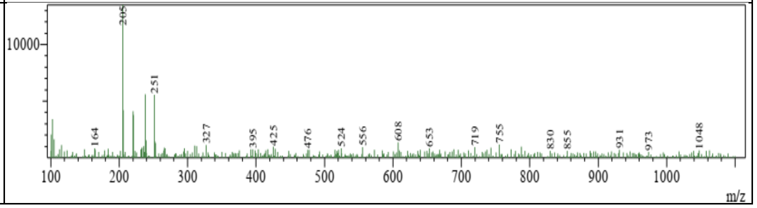

Fragmentation chromatograms resulted from mass fragmentation of LCMS peaks of Fraction 1 and 2 of PPP at each RT as mentioned in Results section

References

1 Saravanan, A. *et al*. Production of pigment using *Aspergillus tamarii*: New potentials for synthesizing natural metabolites. *Environmental Technology & Innovation* **19**, 100967 (2020).

2 Devi, P., Meera, R., Rajasoundarapandian, P. & Mallayasamy, M. Standardization of Imporal, Vivati and Teerankottai Lehyams. **3**, 68-73 (2015).

3 Savithramma, N., Rao, M. L. & Ankanna, S. Screening of traditional medicinal plants for secondary metabolites. *Int J Res Pharm Sci* **2**, 643-647 (2011).

4 Bansode, T. S. & Salalkar, B. Phytochemical analysis of some selected Indian medicinal plants. (2015).
